# Anticancer Activity of Mannose-Specific Lectin, BPL2, from Marine Green Alga *Bryopsis plumosa*

**DOI:** 10.3390/md20120776

**Published:** 2022-12-13

**Authors:** Jei Ha Lee, Set Byul Lee, Heabin Kim, Jae Min Shin, Moongeun Yoon, Hye Suck An, Jong Won Han

**Affiliations:** Department of Genetic Resources, National Marine Biodiversity Institute of Korea, Seocheon-gun 33662, Repulic of Korea

**Keywords:** mannose-binding lectin, BPL2, *Bryopsis plumosa*, anticancer, lung cancer

## Abstract

Lectin is a carbohydrate-binding protein that recognizes specific cells by binding to cell-surface polysaccharides. Tumor cells generally show various glycosylation patterns, making them distinguishable from non-cancerous cells. Consequently, lectin has been suggested as a good anticancer agent. Herein, the anticancer activity of *Bryopsis plumosa* lectins (BPL1, BPL2, and BPL3) was screened and tested against lung cancer cell lines (A549, H460, and H1299). BPL2 showed high anticancer activity compared to BPL1 and BPL3. Cell viability was dependent on BPL2 concentration and incubation time. The IC_50_ value for lung cancer cells was 50 μg/mL after 24 h of incubation in BPL2 containing medium; however, BPL2 (50 μg/mL) showed weak toxicity in non-cancerous cells (MRC5). BPL2 affected cancer cell growth while non-cancerous cells were less affected. Further, BPL2 (20 μg/mL) inhibited cancer cell invasion and migration (rates were ˂20%). BPL2 induced the downregulation of epithelial-to-mesenchymal transition-related genes (Zeb1, vimentin, and Twist). Co-treatment with BPL2 and gefitinib (10 μg/mL and 10 μM, respectively) showed a synergistic effect compared with monotherapy. BPL2 or gefitinib monotherapy resulted in approximately 90% and 70% cell viability, respectively, with concomitant treatment showing 40% cell viability. Overall, BPL2 can be considered a good candidate for development into an anticancer agent.

## 1. Introduction

Lectin is a carbohydrate-binding protein that can agglutinate erythrocytes and cells by specifically binding to carbohydrate moieties on cells [1]. Owing to its carbohydrate-binding properties, it is often suggested for pharmacological applications such as antiviral, antimicrobial, and anticancer therapeutics [2,3,4].

Cancer has high death rates when compared to other human diseases, with over a million people globally newly diagnosed with the condition every year [5]. Although many researchers and countries have invested in anticancer agents, it remains an intractable disease.

Anticancer agents are most commonly developed from antibodies (based on the human immune system) [6], natural products (primarily secondary metabolites) [7], and proteins or peptides [8]. Chemotherapy is the most commonly used anti-cancer approach; however, it has some limitations, such as toxicity [9]. 

Tumor cells have shown various glycosylation patterns as a common feature, making them distinguishable from non-cancerous cells. Cellular glycosylation mechanisms are associated with physiological and pathological functions [10]. The alteration of glycans on the cancer cell surface affects the invasion and migration of cancer cells. Glycan is also involved in signal transduction, cell adhesion, and cell-substrate interactions [11]. Owing to this, biomarkers for diagnosis have been developed based on the glycosylation pattern of tumor cells [12]. 

Lectin can recognize tumor cells by binding to cell surface-altered carbohydrates [13]. Therefore, some lectins have been studied as diagnostic agents against tumor cells. Because lectins are also regulators of inflammation and the immune response toward tumor cells [10], they have been investigated as anticancer agents [14]. Lectin is a well-known protein that can inhibit tumor growth and induce cancer cell death. In the last several decades, several lectins with anticancer activities have been reported (e.g., ConA) [15]. Mistletoe (*Viscum album*) lectin is a well-known lectin that is effective against various neoplastic cells [16]. It induces apoptosis in colorectal cancer cells [17]. Moreover, RCA-I specifically binds to metastasis-associated cell surface glycans and inhibits cell invasion and migration [18]. 

Many marine algal lectins have been reported to be novel proteins. They are believed to have a unique feature compared with other lectins because marine algae have different carbohydrate complexes [19]. To date, various lectin or hemagglutination activities have been reported from the marine algal group [3,20], with various biomedical applications being proposed [21].

*Bryopsis plumosa* is a well-known coenocyte marine green alga containing abundant lectins involved in algal cell regeneration [22]. To date, *Bryopsis plumosa* lectins (BPLs), which specifically bind to different carbohydrates, have been reported. BPL1, 3, and 4 bind to GlcNAc and GalNAc, while BPL2 binds to D-mannose [22,23,24,25]. All *Bryopsis plumosa* lectins in literature were purified, and their amino acid sequences were determined. To date, the potential anticancer effect of *Bryopsis plumosa* lectin has been suggested [25]; however, few studies confirming this postulation have been reported. 

In this study, the anticancer activity of BPLs (BPL1, 2, and 3) against lung cancer cell lines and their regulation of epithelial-to-mesenchymal transition (EMT) pathway-related genes were demonstrated.

## 2. Results

### 2.1. Preaparation of Bryopsis Plumosa Lectins 

**Figure 1:**
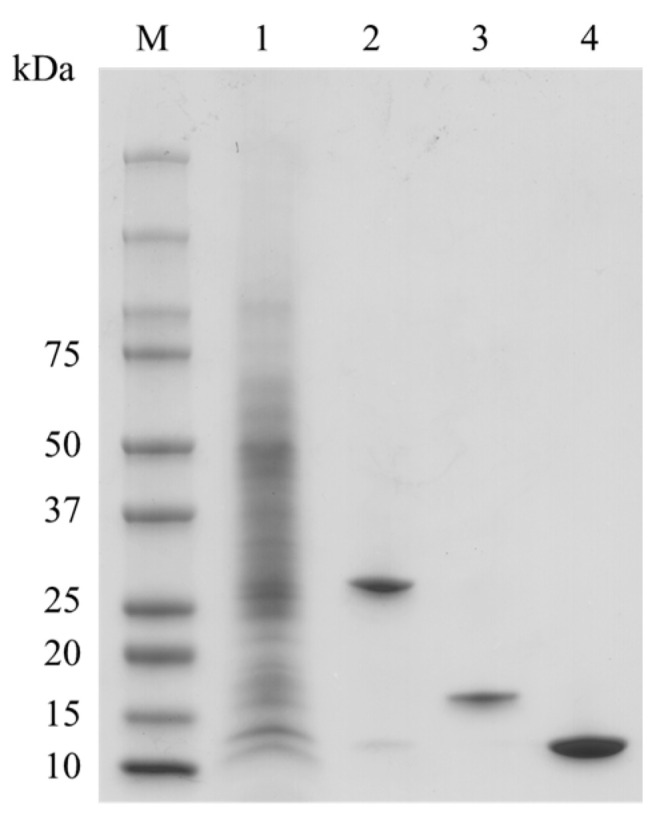
Purification of Bryopsis plumosa lectins (BPLs). M, Molecular weight marker; lane 1, crude extract; lane 2, BPL1; lane 3, BPL2; lane 4, BPL3.

Bryopsis lectin was successfully isolated from the crude extract of Bryopsis plumosa. Combining GalNAc and D-Man affinity chromatography was performed to separate each lectin. Stepwise elution of lectins as different monosaccharides was effective in separating BPL1 and BPL3 (Figure 1). The flow-through fraction contained BPL2, which was successfully isolated using mannose affinity chromatography (Figure 1). The purity of each BPL was more than 95%. All the lectins showed similar hemagglutination activities, as previously reported [22,23,24,25]. 

### 2.2. Cell Viability of Lung Cancer Cells and Non-Cancerous Cells against BPLs

**Figure 2:**
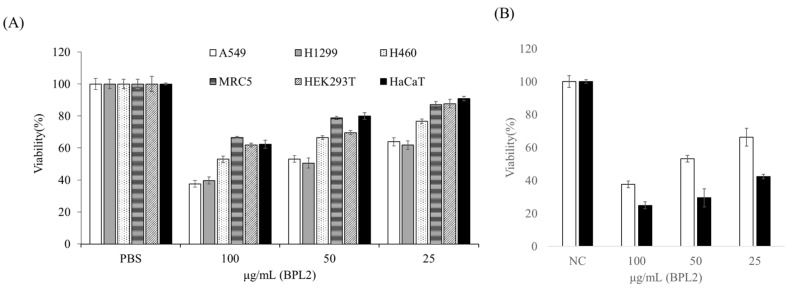
Effect of BPL2 on the viability of cell lines using MTT assays. (**A**) After 24 h of exposure to various cell lines, (**B**) After 24 (white bar) and 72 h (black bar) of exposure to the A549 cell line. Results are represented as the mean ±  standard error.

BPL2 inhibited the viability of lung cancer cell lines, whereas BPL1 and BPL3 did not show the inhibitory activity of the tested cell lines at any concentration (Supplementary Figure S1). Cell viability was dependent on the BPL2 concentration and exposure time. BPL2 activity was most effective in the A549 and H1299 lung cancer cell lines, with cell viability being approximately 40% at 100 μg/mL of BPL2 after exposure for 24 h. However, weak toxicity towards non-cancerous cell lines (MRC5, HEK293T, and HaCaT) was observed at 100 μg/mL (~60–70% viability) (Figure 2A). The increase in the exposure time of BPL2 affected cell viability; cell viability was reduced to approximately 20% after 72 h of exposure (Figure 2B). Based on the cytotoxicity results in non-cancerous cell lines, subsequent experiments were performed at a BPL2 concentration of fewer than 25 μg/mL.

**Figure 3:**
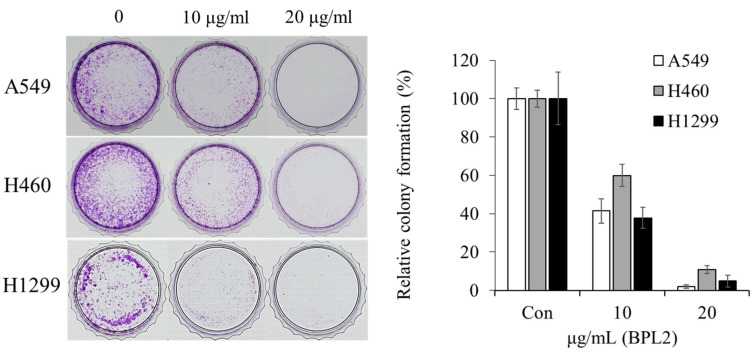
Colony-forming assay for the anticancer activity of BPL2. A549, H460, and H1299 cancer cell lines were used for these studies.

According to the colony-forming assay, BPL2 was effective at low concentrations. All cancer cell lines disappeared after treatment with 20 μg/mL of BPL2. In addition, half of the colonies did not survive at 20 μg/mL BPL2 (Figure 3). 

### 2.3. Determination of Migration and Invasion of Cancer Cells following BPL2 Treatment

**Figure 4:**
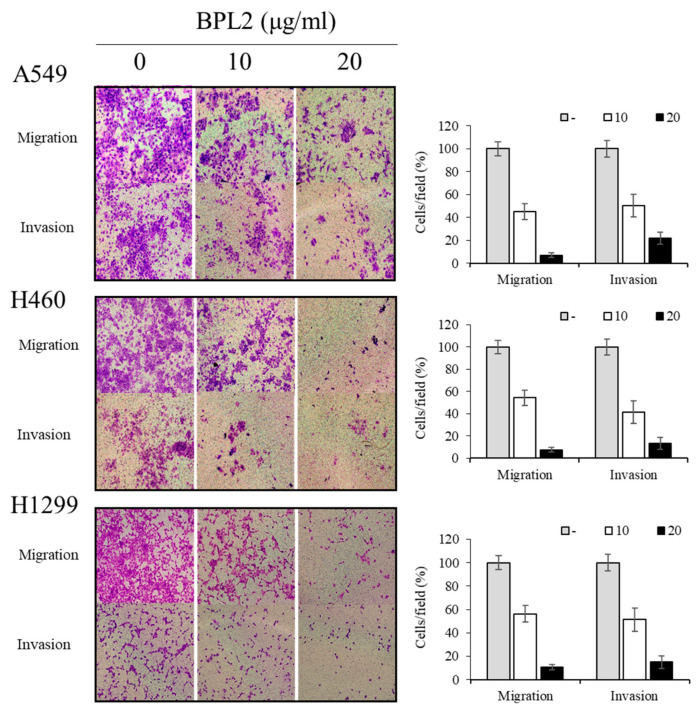
Effect of BPL2 on migration and invasion of cancer cells. Transwell migration (Upper images) and invasion assays (Bottom images) for A549, H460, and H1299 cell lines were performed using different concentrations of BPL2. Representative graphs are shown with the quantification of the randomly selected fields.

BPL2 strongly inhibited the invasion and migration of lung cancer cells in the various cell lines (A549, H460, and H1299). A concentration-dependent effect was observed in both the cell migration and invasion assays. The migration of all cancer cell lines was reduced to less than 10% following treatment with 20 μg/mL of BPL2. The cell invasion rate of lung cancer cells also decreased to 20% at the same treatment conditions (Figure 4).

**Figure 5:**
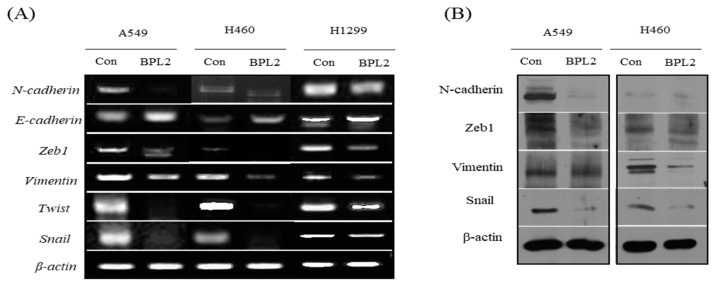
Levels of (**A**) EMT gene and (**B**) protein expression. (**A**) Gene expression in A549, H460, and H1299 cell lines following treatment with BPL2. (**B**) The protein expression in A549 cells following treatment with BPL2. β-actin was used as the control.

The expression levels of EMT-related genes (N-cadherin, E-cadherin, ZEB1, vimentin, and Twist) were investigated to understand the involvement of BPL2 in the EMT pathway. BPL2 induced the downregulation of ZEB1, Twist, vimentin, and N-cadherin and upregulated E-cadherin expression. Gene expression levels showed similar regulation in all tested cancer cell lines, viz. A549, H460, and H1299 (Figure 5A). The levels of EMT-related proteins (N-cadherin, ZEB1, vimentin, and snai1) showed the same trends as their corresponding gene expressions (Figure 5B). 

**Figure 6:**
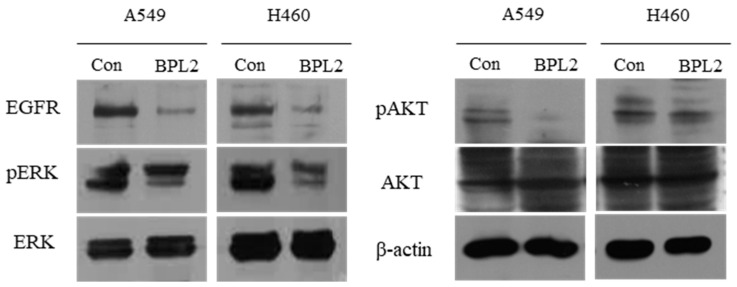
Levels of EGFR signaling-related protein expression. Level of proteins in A549 and H460 cell lines following treatment with BPL2 as determined by Western blot analysis. β-actin was used as the control.

The levels of EGFR signaling-related protein expression were determined. EGFR in A549 and H460 cells decreased significantly upon BPL2 treatment. Activation of ERK and AKT, important downstream targets of the EGFR signaling pathway, reduced significantly (Figure 6).

### 2.4. Analysis for Apoptosis in BPL2-Treated Lung Cancer Cell Lines

**Figure 7:**
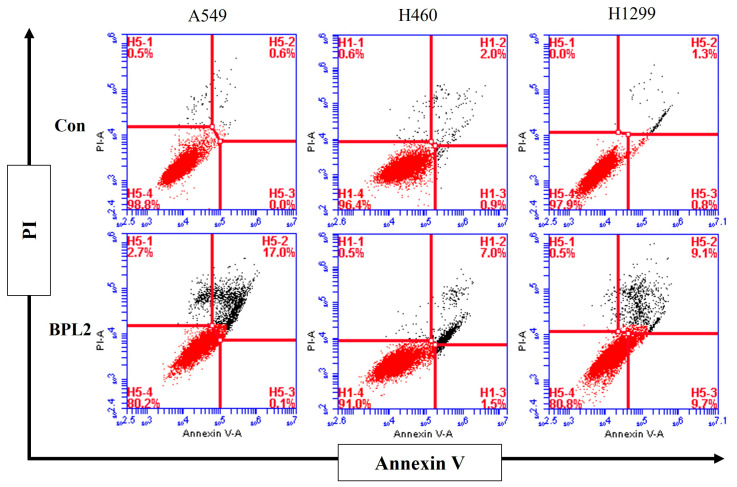
Analysis of apoptosis rates in BPL2-treated lung cancer cell lines. Lung cancer cells were incubated for 48 h with BPL2 and stained with Annexin V/PI for flow cytometric analysis. The upper left panel indicates necrotic cell death; the lower left panel indicates live cells; the upper right panel indicates late apoptosis, and the lower right panel indicates early apoptosis.

To evaluate whether treatment with BPL2 induced apoptosis, an Annexin V/PI staining assay was performed. After treatment with BPL2 at 20 μg/mL for 48 h, significant apoptosis induction was observed. The apoptosis rate increased to 17.1% (early: 0.1%, late: 17.0%) in A549, 9.5% (early: 1.5%, late: 7.0%) in H460, and 18.8% (early: 9.7%, late: 9.1%) in H1299 cells after treatment with BPL2. Necrotic cell death was observed at 2.7% in A549 and 0.5% in H460 and H1299 cells (Figure 7).

### 2.5. Effect of Concomitant Treatment with Gefitinib and BPL2 on Cell Viability

**Figure 8:**
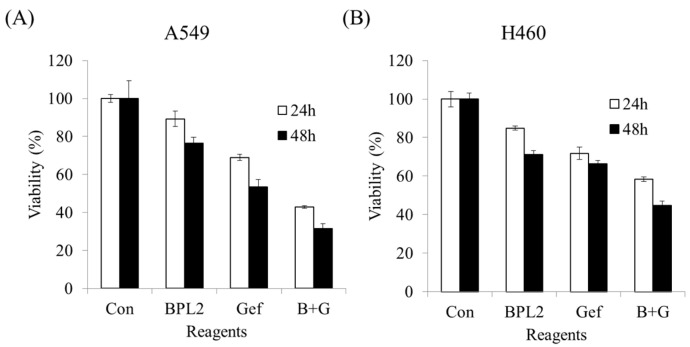
Concomitant treatment or monotherapy using gefitinib (10 μM) and BPL2 (10 μg/mL) on lung cancer cell lines. (**A**) A549, (**B**) H460. Con shows untreated cell lines. B+G shows cells treated with both agents.

Concomitant treatment with gefitinib and BPL2 (10 μM and 10 μg/mL, respectively) showed a synergistic effect on the cancer cell lines compared with monotherapy. BPL2 or gefitinib monotherapy showed approximately 90% and 70% A549 cell viability, respectively, while concomitant therapy showed approximately 40% viability in the same cell line (Figure 8A). H460 cells treated with a combination of gefitinib-BPL2 did not show as much decrease in viability compared to A549 cells (Figure 8B). An increase in incubation time with the treatment agents led to a further 10% reduction in cell viability in both cell lines. 

## 3. Discussion

Similar to all other medicines, a lectin that is to be investigated for its therapeutic activity must be purified from its source [26]. Stepwise purification using two different affinity chromatographic techniques were successful in separating *Bryopsis plumosa* lectins. As previously reported [23,25], both GalNAc binding lectins (BPL1 and BPL3) were isolated by GalNAc affinity chromatography using a two-step elution method, and then the mannose-binding lectin (BPL2) was purified using D-mannose affinity chromatography [25]. The purity of the isolated lectins was sufficient to determine their effectiveness and mechanisms of action in cancer cell lines.

Several anticancer lectins from plants and animals have been reported in recent decades, such as galectin, C-type lectins, sialic acid binding, and Mistletoe lectin [14]. It is known that these lectins recognize carbohydrates on the cell surface and inhibit the survival of cancer cells by various mechanisms. A plant lectin has also been reported to affect apoptosis and autophagy by regulating a signal transduction pathway [14]. The specific binding of lectin to the cancer cell was well reported in targeting and imaging cancer cells. The alternation of cancer cell surface glycan was a well-known phenomenon and the carbohydrate recognition properties of lectin were often applied to cancer cell imaging [27]. Although the direct binding of BPL2 was not determined in this study, the binding of BPL2 on cancer cells could be assumed. The glycan binding specificity of BPL2 has been determined by hemagglutinating inhibition assay and it was specific to the α-methyl-D-mannose (Minimum inhibitory concentration, 3.9 mM), D-mannose (1.9 mM), L-fucose (7.8 mM), and D-glucose (125 mM) [25]. The abundance of high-mannose N-glycan or fucosylated on cancer cells has been reported [28,29]. Owing to the binding properties, lectins have been suggested as potential therapeutic agents that recognize the high-mannose N-glycans occurring at the membrane of various cancer cells [29]. Therefore, mannose-specific algal lectins such as Bryopsis lectin may have anti-cancer and anti-viral activity [29,30].

As expected, the D-mannose-specific lectin, BPL2, showed anticancer activity. However, GlcNAc- and GalNAc-binding lectins did not show any anticancer effects. It may be assumed that different lectins show specific cytotoxic effects against certain cancer cell lines and that the latter two lectins could have anticancer activity in other cancer cells (e.g., cutaneous cancer). The specificity of lectins for distinct cancer cells is a well-known phenomenon. Tian et al. reported the binding affinity and specificity of 27 different lectins in four distinct colorectal cancer cell lines. In addition to the different interactions of 27 lectins with colorectal cancer cell lines, the same lectin displayed differences in four distinct cell lines [31].

The viability of lung cancer cell lines was greatly decreased after exposure to BPL2. The IC_50_ value was approximately 50 μg/mL for A549 and H1299 cells. This is quite a low concentration compared with that of the red alga *Kappaphycus striatus* lectin, KSL, which was reported to have an IC_50_ value in the range of 0.80–1.94 µM (0.22–0.54 mg/mL) [32]. Owing to the cytotoxicity of BPL2 against non-cancerous cell lines, the minimum concentration of BPL2 (˂20 μg/mL) for other experiments was determined based on the cell viability results that did not show cell toxicity. 

Although the mechanisms underlying the anticancer activity of BPL2 were unclear, the molecule clearly showed effective inhibition of cell growth in a colony-forming assay at a low concentration (20 μg/mL). The reduction in cell growth may have been mediated by binding to the surface carbohydrates of cancer cells and inducing cytotoxicity. Mannose-binding plant lectin from *Remusatia vivipara* exhibits a strong glycan-mediated cytotoxic effect and inhibits the growth and motility of human breast cancer cells [33]. Cancer cells often exhibit alterations in the cell surface of polysaccharides that act as tumor-associated antigens. Lectin recognizes altered cell surface carbohydrates and inhibits cell growth through several mechanisms, such as the reactive oxygen species-dependent pathway [34] and an apoptosis-inducing mechanism [35]. Owing to this, BPL2 may have mechanisms that are similar to those of other plant lectins.

Inhibition of cancer invasion and migration is a priority in cancer therapy because most cancer deaths are caused by metastasis [36]. BPL2 clearly inhibited the invasion and movement of cancer cells in all the tested lung cancer cell lines. In general, cancer cell invasion and migration are affected by several mechanisms. For example, a lectin from *Bandeiraea simplicifolia* seeds (BS-I) inhibited cancer cells, hepatocellular carcinoma, invasion, and migration, mediated by inhibiting the activation of the AKT/GSK-3β/β-catenin pathway [37]. AKT/GSK3β/β-catenin signaling contributes to cell migration and the EMT pathway [38], which affects EMT gene expression patterns. EMT is a program of cells that are vital for embryonic development, wound healing, and the malignant progression of cancer [39]. Three of the EMT marker genes, viz. zinc-finger E-box binding protein 1 (ZEB1), vimentin, and Twist, among the reported genes (i.e., ZEB1, Snail, and Twist) for the EMT marker [40] were selected, and the gene expression patterns after or without exposure to BPL2 were determined. All the analyzed genes in the three tested lung cancer cell lines were downregulated following treatment with BPL2, which corresponds to cell migration and invasion experiments. The protein expression at the same conditions correlated significantly with the corresponding gene expression level, although, for vimentin, the trend was unclear. Therefore, the gene expression could be assumed to reflect the corresponding protein levels. 

ZEB1 is a well-known transcription factor that is upregulated in various tumor cell lines and is related to the invasion and migration of cells in patients with lung cancer [41]. It is also a critical regulator of cell plasticity, DNA damage, cancer cell differentiation, and metastasis [42]. BPL2 suppresses ZEB1 gene regulation in lung cancer cell lines and induces cell death. Signal transduction and activation of ZEB1 in EMT plays an important role in embryonic development and malignant progression. It is also associated with resistance to cancer therapies [43]. Suppression of ZEB1 gene expression decreases cancer angiogenesis while eliciting continuous cancer vascular normalization [44]. BPL2 diminished ZEB1 expression, and it could be assumed that it inhibits cancer cell migration via the same mechanisms.

Twist and snail, key transcription factors, are involved in the EMT pathway and play an essential role in cell migration, invasion, and metastasis [45,46]. Although a slight difference was observed among the tested cancer cell lines, downregulation of the Twist and snail genes was clearly defined. Therefore, we assumed that the anticancer ability of BPL2 was related to the inhibition of the EMT pathway. 

Vimentin expression is affected by the downregulation of ZEB1, in turn constraining tumor migration [47,48]. The regulation of Twist is also associated with the expression of membrane proteins (N-cadherin, fibronectin, and vimentin) involved in cell adhesion in cancer cells [49]. Because of the downregulation of transcription factors ZEB1 and Twist, a reduction in vimentin expression after treatment with BPL2 was expected. The expression of vimentin was reduced in the tested cell lines, although different expression levels were observed in each tested cell line. The membrane protein vimentin is widely distributed in the fibroblasts, white blood cells, and vascular endothelial cells. It supports cell membranes and organelles, and a lack of vimentin induces cell migration. BPL2 appears to affect the transcription factor of EMT, disturbs vimentin expression, and ultimately inhibits cancer cell growth, invasion, and migration. Therefore, BPL2 appears to be a candidate inhibitor of the EMT pathway. However, the mechanisms of BPL2 in cancer cell lines are still unclear, whether it is directly or indirectly related; therefore, further comprehensive studies are required to understand the inhibition mechanisms.

The regulation of N-cadherin and E-cadherin is switched during EMT signaling by a complex network of signaling pathways and transcription factors. Downregulation of E-cadherin is often observed in malignant epithelial cancers and is accepted as a tumor suppressor. In contrast to E-cadherin, N-cadherin is downregulated in tumor cells [50]. Similar to the regulation of N-cadherin and E-cadherin in the inhibition of tumor cell lines, treatment with BPL2 led to the upregulation of E-cadherin and downregulation of N-cadherin. The results of the EMT pathway involving marker gene regulation following treatment with BPL2 were well aligned with the suppression of the EMT pathway in tumor cell lines.

Cell surface glycan alteration during the EMT process has been observed in various cancer models. It has been reported that modification of the glycan on the cell surface plays a pivotal role in metastasis [51]. 

The mannan-binding lectin in the reduction of EMT has been reported to be related to the calcium entry machinery [52]. BPL2 does not require a divalent ion for its activity [25]; therefore, it could be assumed that BPL2 is not associated with calcium channels. There are few reports on the involvement of lectin in the EMT pathway. Although BPL2 has not been confirmed to directly contribute to the suppression of the EMT pathway, it could be assumed to inhibit the migration of cancer cells by recognizing the cell surface glycan alternations (high-mannose N-glycan) on cancer cells with metastatic ability.

Gefitinib, an epidermal growth factor receptor tyrosine kinase inhibitor (EGFR-TKI), is a well-known drug used for the treatment of non-small cell lung cancer [53]. Concurrent treatment with anticancer agents to attain therapeutic success is accepted as a common regimen. Simultaneous treatment with BPL2 and gefitinib resulted in synergistic effects. We confirmed that the level of total EGFR expression decreased following BPL2 treatment. BPL2 decreased the activation of ERK and AKT in A549 and H460 cells, followed by the downregulation of cellular EGFR levels. Similarly, a study reported decreased expression of EGFR by lectin protein from *Pseudomonas fluorescens* in gastric cancer cells [54,55]. Consistently, BPL2 significantly reduced the expression of EGFR along with the activation of ERK and AKT, downstream of the EGFR signaling pathway in lung cancer cells. Similarly, the synergistic effect of a combination treatment of gefitinib and docetaxel in EGFR-TKI-sensitive cells has been reported [56].

*Polygonatum odoratum* lectin elicits apoptosis and autophagy in cancer cells. Apoptosis is induced by the Akt-NF-κB pathway in lung cancer cells [57], and the EGFR-mediated Ras-Raf-MEK-ERK pathway in breast cancer cells [55]. Similar to *Polygonatum odoratum* lectin, BPL2 treatment resulted in differential expression of EGFR and EMT pathway-related proteins. Based on the results, it could be concluded that BPL2 could induce apoptosis by similar mechanisms.

The main role of gefitinib is to inhibit tyrosine kinase, involved in cellular proliferation [58] and promotes apoptosis [59]. Based on the results of the Annexin V/PI staining assay, BPL2 was found to induce apoptosis rather than necrosis in lung cancer cell lines. Like BPL2, induction of apoptosis by lectin has been reported, like mistletoe lectin [14,60], a lectin from *Dioclea lasiocarpa* [61], and lectin from *Sophora flavescens* [62]. The synergetic effect may have led to the induction of apoptosis. The combination of mistletoe lectin with other compounds showed a synergistic anti-cancer effect in breast cancer cells [63]. 

The anticancer activity of BPL2 was determined in this study, and it was related to the inhibition of the EMT pathway and induction of apoptosis. Furthermore, concurrent treatment with another anticancer agent, gefitinib, showed a synergistic effect in two lung cancer cell lines (A549 and H460). Therefore, the mannose-binding lectin, BPL2, could be a good candidate for drug development in anticancer therapeutics. 

## 4. Materials and Methods

### 4.1. Preparation of Bryopsis plumosa Lectins (BPLs)

*Bryopsis plumosa* cultured in our laboratory was used to extract BPLs. BPLs were isolated following a previously reported method by Han et al. [23,24,25]. *Bryopsis plumosa* was harvested and washed with 1× Tris-buffered saline (TBS, pH 7.5) containing 1 mM CaCl_2_ and 1 mM MgCl_2_. The harvested samples were ground into a fine powder, after exposure to liquid nitrogen, using a mortar and pestle. Five volumes of ice-cold 1× TBS were added to the ground samples and incubated for 3 h at 4 °C. The incubated sample was centrifuged at 25,000× *g* for 30 min at 4 °C, and the cell debris was removed. The crude extract was loaded directly onto an affinity chromatography column. A Bio-Rad NGC FPLC system (Bio-Rad Laboratories, Hercules, CA, USA) was used for chromatographic analysis at a flow rate of 1 mL/min. First, GalNAc-agarose was used to purify BPL1, BPL3, and BPL4 using a stepwise elution method. The affinity column was washed with 10 volumes of 1× TBS. BPL3 and BPL4 were eluted using 0.2 M of GlcNAc in 1× TBS and then BPL1 was eluted using 50 mM GalNAc in 1× TBS. The flow-through from GalNAc-agarose, which contained mannose-binding lectin, was loaded onto the mannose-agarose. BPL2 was eluted with 0.5 M D-mannose dissolved in 1× TBS.

All lectins were confirmed using SDS-PAGE and UV spectrophotometry. The BPLs were dialyzed using 1× PBS and stored at −20 °C until use.

### 4.2. Cell Culture and Viability Assay (Determination of Viability of Tumo Cells)

Metastatic lung cancer cell lines (A549, H460, and H1299) were used to determine tumor cell viability. Non-cancerous and immortalized cells (MRC5, HEK293T, and HaCaT cells) were used as controls (Supplementary Table S1). All cells used for the cell viability test were cultured at 37 °C under atmospheric conditions of 5% CO_2_ in fetal bovine serum (FBS) containing antibiotics (penicillin and streptomycin). The growth rate of the cells was determined by CCK-8 analysis. Aliquots of each cell line were added to 96-well plates to achieve a cell number of 5 × 10^3^ cells per well and incubated in a 5% CO_2_ atmosphere at 37 °C for 24 h. BPLs (BPL1, 2, and 3) at concentrations of 25, 50, and 100 μg/ml, respectively, were added to the cells and then incubated for 24–72 h. After incubation, the culture medium was removed and the cells were incubated in a fresh culture medium containing CCK-8 solution for 3 h. Cell viability was determined by measuring absorbance at 450 nm using a 96-well plate reader (Spectramax i3x; Molecular Devices, San Jose, CA, USA). Live cells were calculated as a percentage. All experiments were repeated at least three times.

### 4.3. Determination of Cell Growth Rate Based on BPL Treatments 

The cell lines were cultured in the same manner as for the cell viability test. A colony-forming test was performed to compare the growth rate among cell lines treated with different concentrations of BPLs. Each cell line was divided into 1 × 10^3^ cells per 30 mm dish and cultured at 37 °C in a CO_2_ incubator for 24 h. The cell lines were treated with BPLs at concentrations of 10 and 20 μg/mL. After being cultured for 7 days, the culture medium was discarded and stained with 0.5% crystal violet solution for 10 min. The stained cells were washed several times with 1× PBS and the growth rate was observed under a microscope.

### 4.4. Determination of Migration and Invasion of Cancer Cells

The migration and invasion assays were performed using a Transwell (Falcon, BD labware, Bedford, MA, USA) with a 0.8 μm pore size. The EMT protein marker was used to analyze the migration ability. The lung cancer cell lines were incubated at 37 °C for 48 h after inoculation into the migration well to obtain a density of 1 × 10^5^ cells/well, which were stained using crystal violet solution.

### 4.5. Comparison of the Expression Level of Cell Migration and Invasion-Related Genes, and EGFR-Related Proteins 

The expression levels of cancer cell-related genes (ZEB1, vimentin, and Twist) were analyzed using RT-PCR. Total RNA from each cell was isolated using the TRIzol RNA extraction solution. RNA quality was determined by gel electrophoresis using a UV-spectrophotometer. First-strand cDNA was synthesized using the Transcriptor First Strand cDNA Synthesis Kit (Roche Diagnostics, Penzberg, Germany). One microgram of total RNA was used for first-strand cDNA synthesis. Primer information for the RT-PCR is listed in Supplementary Table S2. Amplification was performed using an Applied Science PCR machine under the following conditions: pre-denaturation at 94 °C for 5 min, 30 cycles of denaturation at 94 °C for 5 min, annealing at 56 °C for 1 min, extension at 72 °C for 1.5 min; and final extension at 72 °C for 5 min. The relative expression levels of target genes were analyzed by gel electrophoresis.

For analysis of EMT-related protein levels, Western blot analysis was performed. Cells were lysed in a buffer with protease inhibitor cocktails (Sigma-Aldrich). Protein concentrations were determined by the Bradford assay (Bio-Rad, Hercules, CA, USA). Equal amounts of protein were separated on 10% sodium dodecyl sulfate-polyacrylamide gel (SDS-PAGE) and transferred onto NC membranes. Membranes were incubated with each antibody in a blocking solution overnight. After washing with Tris-buffered saline, membranes were incubated with mouse secondary antibody (Abcam, Cambridge, MA, USA) and visualized using a Supersignal west atto ultimate sensitivity substrate (Thermo Scientific, A38555). Antibodies specific for N-cadherin (59987), ZEB1 (515797), Vimentin (6260), Snai1 (271977), and β-actin (47778) were purchased from Santa Cruz Biotechnology.

The analysis of EGFR-related proteins was performed by the same procedure as for EMT-related proteins. The antibodies for EGFR (377547), pERK (7383), ERK (514302), AKT (5298), and pAKT (271966) were obtained from Santa Cruz Biotechnology.

### 4.6. The Effect of Concurrent Treatment (Gefitinib and BPL2) on Lung Cancer Cell Viability

Human cancer cell lines (A549 and H460) were used to determine the effect of concomitant drug administration. Cell lines were prepared following the method described above for cell viability. Concomitant gefitinib-BPL2 or BPL2 (10 μg/mL) and gefitinib (10 μM) monotherapy were used to treat the cell lines. The treated cancer cell lines were incubated for 24 or 48 h, and viability was measured using CCK-8 analysis kits. 

### 4.7. Flow Cytometric Analysis for Apoptosis in BPL2-Treated Lung Cancer Cells

A549 and H1299 cell lines were treated with 20 μg/mL of BPL2 and incubated for 48 h in a CO_2_ incubator. The cells were double stained using the AnnexinV/PI apoptosis detection kit (556547; BD Biosciences, San Jose, CA, UAS) following the manufacturer’s instructions. Apoptosis was determined using a flow cytometer (Accuri C6 Plus; BD Biosciences.

## 5. Conclusions

The anticancer activity of algal lectins has been studied for several decades. Lectins from *Bryopsis plumosa* have been suggested as candidate antitumor agents. Herein, the anticancer activity of BPL2 was demonstrated in lung cancer cell lines, and the inhibition of cell migration and invasion by BPL2 was presumed to be related to the EMT pathway. Concurrent treatment with BPL2 and gefitinib had a synergetic effect on investigated lung cancer cell lines. Therefore, BPL2 could be a good candidate anticancer agent for lung cancer therapy. 

## Supplementary Materials

The following are available online at https://www.mdpi.com/article/10.3390/md20120776/s1: Table S1: Cancer and non-cancerous cell lines used for the anticancer assay, Table S2: Primer sequences used for RT-PCR analyses. Figure S1: Effect of BPL on the viability of A549 and H460 cell lines using MTT assays.
