# Supplementary material for: Anticancer Activity of Mannose-Specific Lectin, BPL2, from Marine Green Alga Bryopsis plumosa
Source: Mar Drugs. 2022 Dec 13;20(12):776. doi: 10.3390/md20120776 (PMC9788543; doi:10.3390/md20120776)
Supplement: Supplementary file 1 [file marinedrugs-20-00776-s001.zip › marinedrugs-2047509-supplementary.pdf]

marine drugs

MDPI

Article

Anticancer activity of mannose-specific lectin, BPL2, from marine green alga *Bryopsis plumosa*

Jei Ha Lee, Set Byul Lee, Heabin Kim, Jae Min Shin, Moongeun Yoon, Hye Suck An, and Jong Won Han*

Department of Genetic Resources, National Marine Biodiversity Institute of Korea, Seocheon-gun 33662, Republic of Korea

* Correspondence: jwhan@mabik.re.kr; Tel.: (+82 419500913)

Supplementary Figure S1. Effect of BPL on the viability of A549 and H460 cell lines using MTT assays

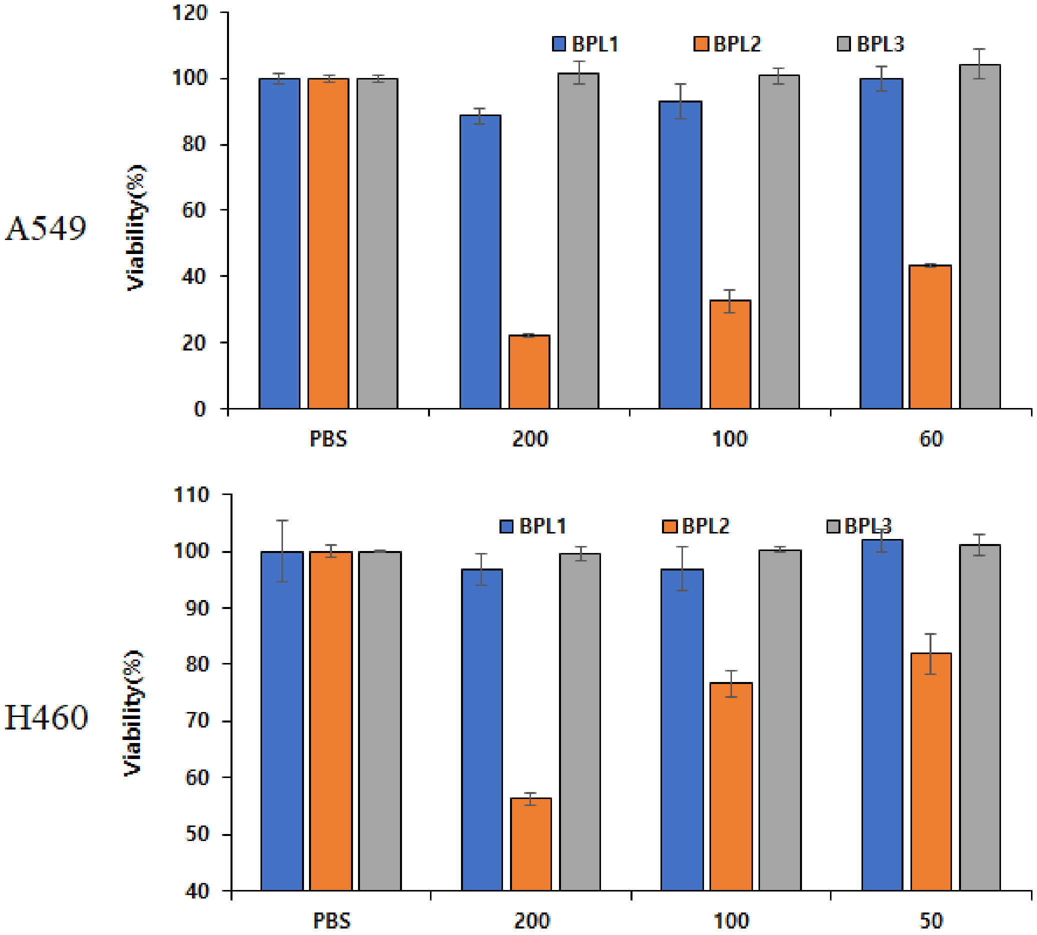

Supplementary Table S1. Cancer and normal cell lines used for the anticancer assay

Cell line	Tissue	Histology/ Derived from	Invasivity
A549	Lung	Adenocarcinoma, primary	++
H460	Lung	Large, Pleural effusion	+
H1299	Lung	Carcinoma, Lymph node metastasis	+++
MRC-5	Lung	Normal fibroblast	-
293T	Kidney	Embryonic kidney	-
HaCaT	Keratinocyte	Normal keratinocyte	-

Supplementary Table S2. Primer sequences used for RT-PCR analysis

Primer name	Sequence (5′-3′)
N-cad-fw	ACTTGCCAGAAAACTCCAGG
N-cad-rv	TGGTGTATGGGGTTGATCCT
E-cad-fw	TGGATAGAGAACGCATTGCC
E-cad-rv	AAAATCCAAGCCCGTGGTG
Zeb1-fw	CGGCGCAATAACGTTACAAA
Zeb1-rv	AAAGGTGTAACTGCACAGGG
Vimentin-fw	GAGAACTTTGCCGTTGAAGC
Vimentin-rv	TCTGCTGGTATATGAGTGCTG
Twist-fw	CTGGACTCCAAGATGGCAAG
Twist-rv	AGAATGCAGAGGTGTGAGGA
GAPDH-fw	GACAGTCAGCCGCATCTTCT
GAPDH-rv	GCGCCCAATACGACCAAATC
